# Impaired health-related quality of life in long-COVID syndrome after mild to moderate COVID-19

**DOI:** 10.1038/s41598-023-34678-8

**Published:** 2023-05-12

**Authors:** Stefan Malesevic, Noriane A. Sievi, Patrick Baumgartner, Katharina Roser, Grit Sommer, Dörthe Schmidt, Florence Vallelian, Ilijas Jelcic, Christian F. Clarenbach, Malcolm Kohler

**Affiliations:** 1 Faculty of Medicine, University of Zurich, Zurich, Switzerland; 2 Department of Pulmonology, University Hospital Zurich, 8091 Zurich, Switzerland; 3 Department of Cardiology, University Hospital Zurich, Zurich, Switzerland; 4 Department of Internal Medicine, University Hospital Zurich, Zurich, Switzerland; 5 Department of Neurology, University Hospital Zurich, Zurich, Switzerland; 6 Department of Health Sciences and Medicine, University of Lucerne, Lucerne, Switzerland; 7 Division of Pediatric Endocrinology, Diabetology and Metabolism, Department of Pediatrics, Inselspital, Bern University Hospital, University of Bern, Bern, Switzerland; 8 Department of Biomedical Research, University of Bern, Bern, Switzerland

**Keywords:** Public health, Quality of life

## Abstract

A growing number of patients with SARS-CoV-2 infections experience long-lasting symptoms. Even patients who suffered from a mild acute infection show a variety of persisting and debilitating neurocognitive, respiratory, or cardiac symptoms (Long-Covid syndrome), consequently leading to limitations in everyday life. Because data on health-related quality of life (HRQoL) is scarce, we aimed to characterize the impact of Long-Covid symptoms after a mild or moderate acute infection on HRQoL. In this observational study, outpatients seeking counseling in the interdisciplinary Post-Covid consultation of the University Hospital Zurich with symptoms persisting for more than 4 weeks were included. Patients who received an alternative diagnosis or suffered from a severe acute Covid-19 infection were excluded. St. George’s Respiratory Questionnaire (SGRQ), Euroquol-5D-5L (EQ-5D-5L), and the Short form 36 (SF-36) were distributed to assess HRQoL. 112 patients were included, 86 (76.8%) were female, median (IQR) age was 43 (32.0, 52.5) years with 126 (91, 180) days of symptoms. Patients suffered frequently from fatigue (81%), concentration difficulties (60%), and dyspnea (60%). Patients mostly stated impairment in performing usual activities and having pain/discomfort or anxiety out of the EQ-5D-5L. EQ index value and SGRQ activity score component were significantly lower in females. SF-36 scores showed remarkably lower scores in the physical health domain compared to the Swiss general population before and during the COVID-19 pandemic. Long-Covid syndrome has a substantial impact on HRQoL. Long-term surveillance of patients must provide clarity on the duration of impairments in physical and mental health.

*Trial registration:* The study is registered on www.ClinicalTrials.gov, NCT04793269.

## Background

So far, more than 750 million people have been diagnosed with Covid-19 disease following a pandemic of the novel coronavirus, SARS CoV-2^1^. In Switzerland, up to March 2023 there were over 14,000 deaths due to SARS CoV-2^2^. Yet, the vast majority of infected people experience mild to moderate symptoms, omitting hospitalization and/or mechanical ventilation^3^. Although long-term health consequences and persistent symptoms were described in hospitalized patients^4^, there is growing evidence that even patients with a mild or moderate acute infection suffer from persisting symptoms or develop new, long-lasting symptoms after initial recovery from infection, termed Long-Covid or Post-Covid-19 syndrome^5,6^. Estimations about the prevalence of Long-Covid syndrome vary broadly, as definitions, time-points of assessments, and recruitment methods differ between studies. Prevalence estimates range from 2.2 up to 86%, depending on the severity of the disease^7,8^. Nonetheless, Long-Covid syndrome affects survivors of Covid-19 at all disease severity scales and has gained attention in the medical as well as political community. Little is known about the pathophysiological mechanisms of Long-Covid syndrome. Thus, the symptoms involving more than one organ in patients with mild acute disease and no signs of persistent tissue damage remains astonishing. The induction of immune activation through SARS-CoV-2 persistence in the body^9–11^ as well as lymphocyte dysfunctions resembling autoimmune disorders^12^ may be possible drivers of Long-Covid syndrome. Studies conducted so far showed a broad range of symptoms frequently consisting of fatigue, headache, dyspnea, and anosmia^13^. Some similar clinical features have already been observed in different corona virus infections (e.g. MERS and SARS-CoV-1)^14,15^. Moreover, chronic fatigue syndrome has been proposed to be triggered by a viral infection among other reasons^16^.

Quality of life is perceived differently between individuals. The World Health Organization (WHO) defined this complex concept as “an individual’s perception of their position in life in the context of the culture and value systems in which they live and in relation to their goals, expectations, standards and concerns”^17^. Clearly, health plays a major role in an individual’s ability to live a fulfilling life. More specifically, quantifying mental and physical health enables health care workers to assess the impact of diseases and individualize further care. Hitherto, many studies showed the extensive impact that chronic conditions contribute to health-related quality of life (HRQoL)^18–21^.

HRQoL is impaired after hospitalization due to Covid-19^22^, but it is unclear how HRQoL is affected in patients suffering from Long-Covid syndrome after a mild or moderate acute infection. A long-lasting reduction in HRQoL after a mild or moderate infection would add even more to the burden of the Covid-19 pandemic, making it all the more important to reinforce prevention strategies and find appropriate therapeutic options.

This study aimed to assess HRQoL in a cohort of Swiss patients suffering from Long-Covid syndrome after a mild or moderate acute infection.

## Method

### Study design and patient population

The University Hospital of Zurich developed an interdisciplinary outpatient clinic for patients suffering from Long-Covid syndrome, which included the departments of Pulmonology, Cardiology, Neurology, and Internal Medicine. Patients were triaged into one of the departments depending on their symptom constellation. Patients received questionnaires regarding HRQoL during the consultation or it was sent to them in hindsight.

Patients who filled out the questionnaires properly and either suffered from Long-Covid syndrome or in whom a Long-Covid syndrome was highly suspected after a mild or moderate acute Covid-19 infection were further evaluated. Long-Covid syndrome was defined according to the UK National Institute for Health and Care Excellence as ongoing symptomsor newly developed symptoms after the acute phase of confirmed or suspected SARS-CoV-2 infection that persist beyond fourweeks from the initial infection and cannot be explained by an alternative diagnosis^23^. Symptoms could have been of systemic nature (e.g. fatigue, recurrent fever) or organ specific (e.g. cough, palpitations, gastrointestinal symptoms). There was no minimum number of symptoms required for the diagnosis and symptoms could have affected more than one organ system. Patients were excluded if a different diagnosis was made by clinical evaluation at outpatient visits explaining their symptom constellation (e.g. hypothyroidism, depressive episode). Moreover, patients were excluded if they required prolonged hospitalization, supplemental oxygen therapy or intensive care treatment during the acute phase of Covid-19 infection, which was termed as severe infection according to the WHO definition^24^.

Information about demographics, symptoms during acute infection as well as Long-Covid symptoms were drawn from systematically documented medical reports. Data on HRQoL assessed with the Short Form-36 version 2 (SF-36v2) in a Swiss cohort at the beginning of the pandemic (CoWell study) were collected by the University of Lucerne, Department of Health Sciences and Medicine, and shared for this study. This study was conducted in accordance with the declaration of Helsinki and all subjects provided written informed consent by general consent. The Ethics Committee of the Canton of Zurich approved the study (BASEC 2021-00280), and the study is registered on www.ClinicalTrials.gov, NCT04793269.

### Questionnaires

#### St. George’s respiratory questionnaire

The St. George’s Respiratory Questionnaire (SGRQ) is a validated disease-specific quality of life assessment tool for obstructive airway diseases^25,26^. It assesses HRQoL from the perspective of respiratory symptoms, which are commonly mentioned by patients suffering from Long-Covid syndrome. The questionnaire consists of two parts measuring symptoms in the first part as well as activity limitation, social and emotional impact of the disease in the second part. Scores range from 0 (no impairment) to a maximum score of 100 (maximum impairment), consequently lower scores meaning poorer quality of life^27^.

#### EuroQol 5 dimension 5 level

The EuroQol 5 Dimension 5 Level (EQ-5D-5L) is a widely used generic measure of health status^28^. In the first part, the descriptive system, the respondent classifies his/her health according to five dimensions (mobility, self-care, usual activities, pain/discomfort, anxiety/depression). Each dimension is ranked into five levels from “no problems” up to “extreme problems”. Each health state can potentially be assigned to a summary index score based on societal preference weights for the health state. Index scores range from less than 0 to 1, where 0 represents a value of a health state equivalent to dead, negative values represent values as worse than dead, and 1 represents full health. For Switzerland, the index scores were calculated using German country value sets, as no value sets for Switzerland are available and we judged the population of Germany to be similar to the Swiss population. In the second part of the questionnaire, patients rate their own perceived health on a visual analog scale (VAS) ranging from 0 (worst health) to 100 (best health).

#### Short form-36

The Short Form Health 36 (SF-36) is the most commonly used, multidimensional instrument for measuring HRQoL^29^. All but one of the 36 questions are assigned to one of eight dimensions of health, namely: physical functioning, role limitations due to physical problems, pain, general health, vitality, social functioning, role limitations due to emotional problems, and mental health. The health dimensions consist of the sum scores of the assigned questions. Transformed scores range from 0 (worst possible health) to 100 (best possible health). Additionally, two summary score components can be calculated, the physical component summary (PCS) and the mental component summary (MCS). After calculating the eight dimension scores, a z-score is determined for each dimension by subtracting the dimension mean of the U.S. or Swiss general population from an individual’s dimension score and dividing it by the standard deviation from the U.S. general population^30^ and Swiss population^31^, respectively. Each of the eight z-scores is multiplied by the corresponding factor scoring coefficient (separately for PCS and MCS) for the dimension^31,32^. Products of the z-scores are summed together, multiplied by 10, and added to 50 to linearly transform PCS and MCS to T-score metric. A value of 50 of the norm based score represents the mean of the respective reference population and higher values mean better HRQoL.

### Statistical analysis

Continuous variables are presented as mean ± standard deviation (SD) in case of a normal distribution and median (interquartile range (IQR)) otherwise. For comparability with existing literature, mean ± SD in not normally distributed data is presented as well. Categorical variables are expressed as number and percentages. Differences between groups were compared using independent sample *t-*test and Mann–Whitney *U* test for continuous variables, and chi-squared test for categorical variables. All statistical tests were two-tailed and a *P* value of < 0.05 was considered as statistically significant. Statistical analysis was performed using Stata version 16.1 (StataCorp. 2019, Texas, TX, USA).

### Ethics approval

This study was conducted in accordance with the declaration of Helsinki. The Ethics Committee of the Canton of Zurich approved the study (BASEC 2021-00280), and the study is registered on www.ClinicalTrials.gov, NCT04793269.

## Results

### Study sample

**Figure 1:**
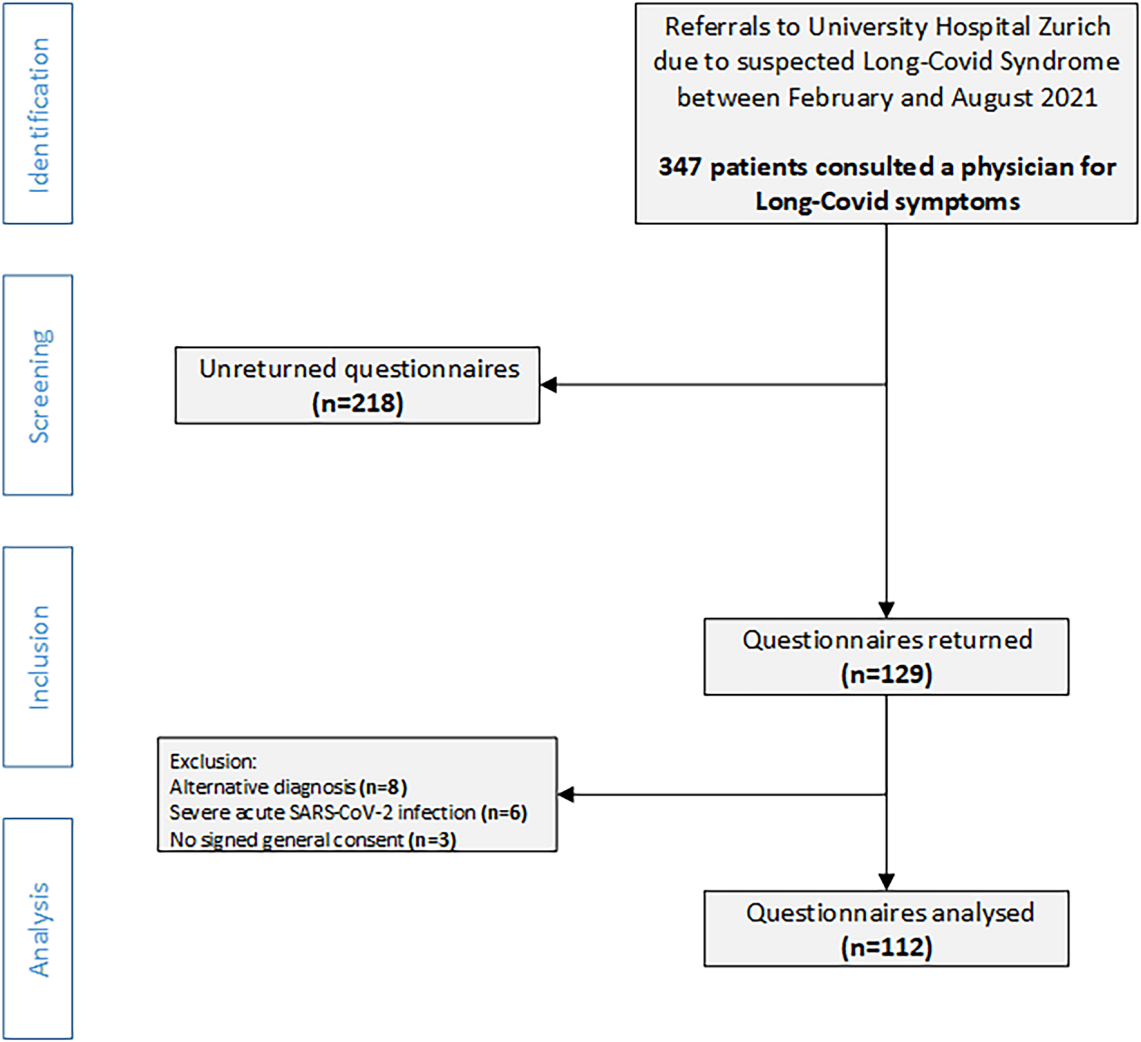
Study flow. Figure was created using MS Office professional plus 2016 Powerpoint.

Between February and August 2021, 112 Long-Covid patients returned HRQoL questionnaires and met all eligibility criteria (Fig. 1). Subjects were predominantly women (76.8%) with a median (IQR) age of 43 (32, 52.5) years. The median (IQR) score for body-mass-index was 24.4 (21.9, 27.8) kg/m2. Fifteen (13.4%) patients suffered from asthma, 15 (13.4%) had prepandemic mental health issues and 28 (25%) had at least one relevant comorbidity. Patients mostly suffered initially from a mild acute SARS-CoV-2 infection (90.2%). Median (IQR) time from diagnosis to the first visit at the University Hospital Zurich was 126 (91, 180) days and median (IQR) time from symptom onset until completion of questionnaires was 153 (114, 211) days. One third of the patients stated to have reduced workload. Further patient characteristics are presented in Table 1.

**Table 1:** Patient characteristics of Long-Covid cohort.

Patient characteristics	
N	112
Sex	
Female	86 (76.8)
Male	26 (23.2)
Age, years (median, IQR)	43 (32.0, 52.5)
Body mass index, kg/m^2^ (median, IQR)	24.4 (21.88, 27.78)
WHO classification regarding acute Covid-19 infection	
Mild	101 (90.2)
Moderate	11 (9.8)
Time from diagnosis to first visit, days (median, IQR)	126 (91, 180)
Time from symptom onset to filling out questionnaires, days (median, IQR)	153 (114, 211)
Smoking history	
Current	15 (13.4)
Former	27 (24.1)
Never	59 (52.7)
Nationality	
Swiss	81 (72.3)
Foreigner	23 (20.5)
Unknown	8 (7.2)
Ethnicity	
Caucasian	96 (85.7)
Not caucasian	2 (1.8)
Unknown	14 (12.5)
Marital status	
Married or partnership	55 (49.1)
Single	15 (13.4)
Unknown	42 (37.5)
Reduced employment due to long-covid syndrome	
Yes	37 (33)
Reduced ≥ 50%	16 (14.3)
Reduced < 50%	19 (17)
No	25 (22.3)
Comorbidities	
Asthma	15 (13.4)
Prepandemic mental health	15 (13.4)
Other relevant comorbidities^a^	28 (25)

Values are N (%) unless otherwise stated.

^a^Other relevant comorbidities were relevant cardiovascular disorders, rheumatological diseases and diseases of the thyroid.

Participant characteristics were comparable regarding sex and age between our study and the CoWell study cohort, whereas the pre-pandemic study cohort of the Swiss general population showed a slightly lower proportion of female participants (76.8% vs. 76% vs. 58.1%) and were slightly older (mean age of 43 years vs. 45 years vs. 49 years).

### Symptom characterization

Most frequently, patients reported neuropsychiatric symptoms such as fatigue (81.3%) and concentration difficulties (59.8%) followed by cardio-respiratory symptoms such as dyspnea (59.8%), performance intolerance (52.7%), and thoracic pain (49.1%). A considerable portion of patients suffered from persistent loss of smell (31.3%) or persistent loss of taste (20.5%). Table 2 contains all symptom frequencies.

**Table 2:** Symptom characterization of Long-Covid cohort.

Symptom	N (%)
Fatigue	91 (81.3)
Concentration difficulties	67 (59.8)
Dyspnea	67 (59.8)
Performance intolerance	59 (52.7)
Thoracic pain	55 (49.1)
Headache	46 (41.7)
Smell alteration	35 (31.3)
Cough	32 (28.6)
Sleeping difficulties	32 (28.6)
Muscle pain	31 (27.7)
Dizziness	31 (27.7)
Muscle weakness	30 (26.8)
Palpitations	28 (25.0)
Weight loss	25 (22.3)
Taste alteration	23 (20.5)
Weight gain	18 (16.0)
Memory difficulties	17 (15.1)
Gastrointestinal symptoms (either nausea, vomiting or diarrhea)	17 (15.1)
Sputum production	14 (12.5)
Reflux	8 (7.1)
Temperature	6 (5.4)
Joint pain	5 (4.5)
Orthopnea	4 (3.6)
Swallowing difficulties	3 (2.7)
Postexertional malaise	3 (2.7)
Tingling paresthesia	4 (3.6)
Hair loss	1 (0.9)

### SGRQ questionnaire

Overall, the median (IQR) SGRQ scores for the symptom component, activity component, impacts component, and total component were 42.7 (28.0, 57.0), 59.0 (32.5, 72.8), 25.7 (13.3, 41.7) and 39.6 (26.7, 54.3), respectively (Table 3). There was a total of 3 (2.75%) missing scores in the symptom component, 16 (16.7%) in the activity component, 11 (10.9%) in the impact component, and 23 (25.8%) in the total component of the SGRQ scores. A subgroup analysis can be found in Table S1 of the supplemental material. Females had significantly greater impairment in the activity component (*p* = 0.005). There was no difference between mild or moderate acute infections regarding the different SGRQ component scores.

**Table 3:** SGRQ component scores, EQ-5D-5L index and VAS scores.

	Median (IQR)	Mean (SD)
SGRQ symptom score	42.7 (28.0, 57.0)	NA
SGRQ activity score	59.0 (32.5, 72.8)	NA
SGRQ impact score	25.7 (13.3, 41.7)	NA
SGRQ total score	39.6 (26.7, 54.3)	NA
EQ-5D-5L index scores	0.806 (0.578, 0.909)	0.738 (0.21)
EQ-5D-5L VAS	62.9 (40, 75)	59.0 (20.3)

*SGRQ* St. George’s Respiratory Questionnaire (higher scores indicating lower health-related quality of life), *EQ-5D-EL* Euroquol 5 Dimension 5 Levels (higher scores indicating greater health-related quality of life), *VAS* Visual Analogue Scale (higher scores indicating greater health-related quality of life), *IQR* interquartile range, *SD* standard deviation, *NA* not available.

### EQ-5D-5L questionnaire

**Figure 2:**
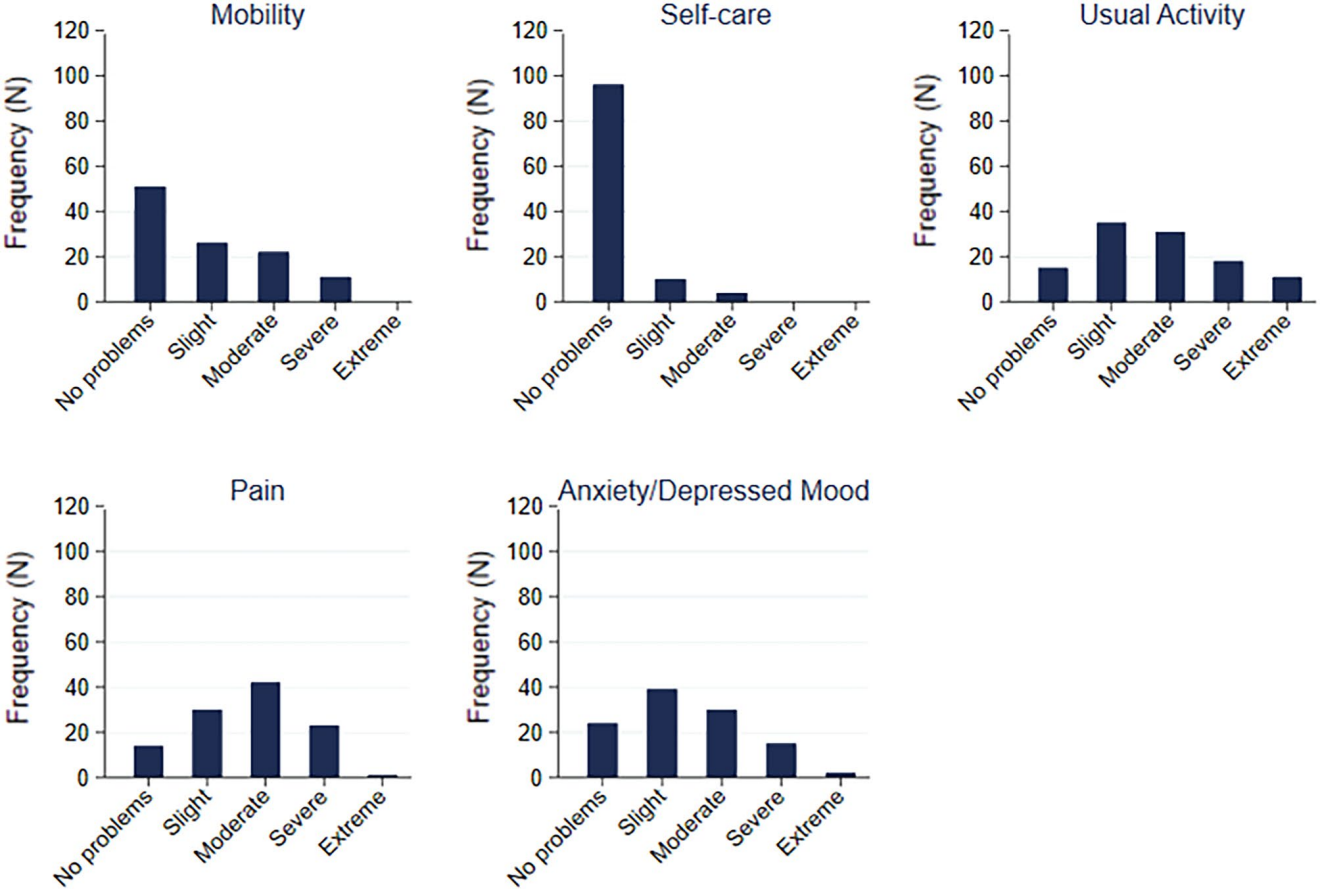
Distribution of EQ-5D-5L dimension responses. Figure shows the level of impairment frequency (n = 110) in the sub-dimensions mobility, self-care, usual activities, pain/discomfort and anxiety/depression. Rating goes from “no problems” to “extreme problems”. Frequently reported moderate to extreme impairments are shown in the sub-dimensions usual activities, pain/discomfort and anxiety/depression**.** Figure was created using Stata version 16.1 (StataCorp. 2019, Texas, TX, USA, https://www.stata.com/products).

When describing the distribution of EQ-5D-5L dimension responses, patients suffering from Long-COVID symptoms were more likely to develop problems in the dimensions “usual activities”, “pain/discomfort” and “anxiety/depression” (Fig. 2 and Table S5 in supplemental material). Most of the participants had no problems with self-care, a few reported moderate problems. There were 11 (10%) participants who were unable to do usual activities. Around half of the patients did not report mobility problems, whereas the other half reported having slight to severe problems.

Regarding EQ-5D-5L VAS scores, the median (IQR) value was 62.9 (40, 75) with 8 patients (7.3%) between 0 and 25, 29 (26.4%) between 26 and 50, 51 (46.4%) between 51 and 75, and 22 patients (20.0%) between 76 and 100. Further results on EQ-5D-5L are shown in Table 3.

Median (IQR) value for the index score was 0.806 (0.578, 0.909). A subgroup analysis (Table S2, supplemental material) showed statistically significant lower scores in females (*p* < 0.001) and no difference in disease severity was detected.

### SF-36 questionnaire

**Figure 3:**
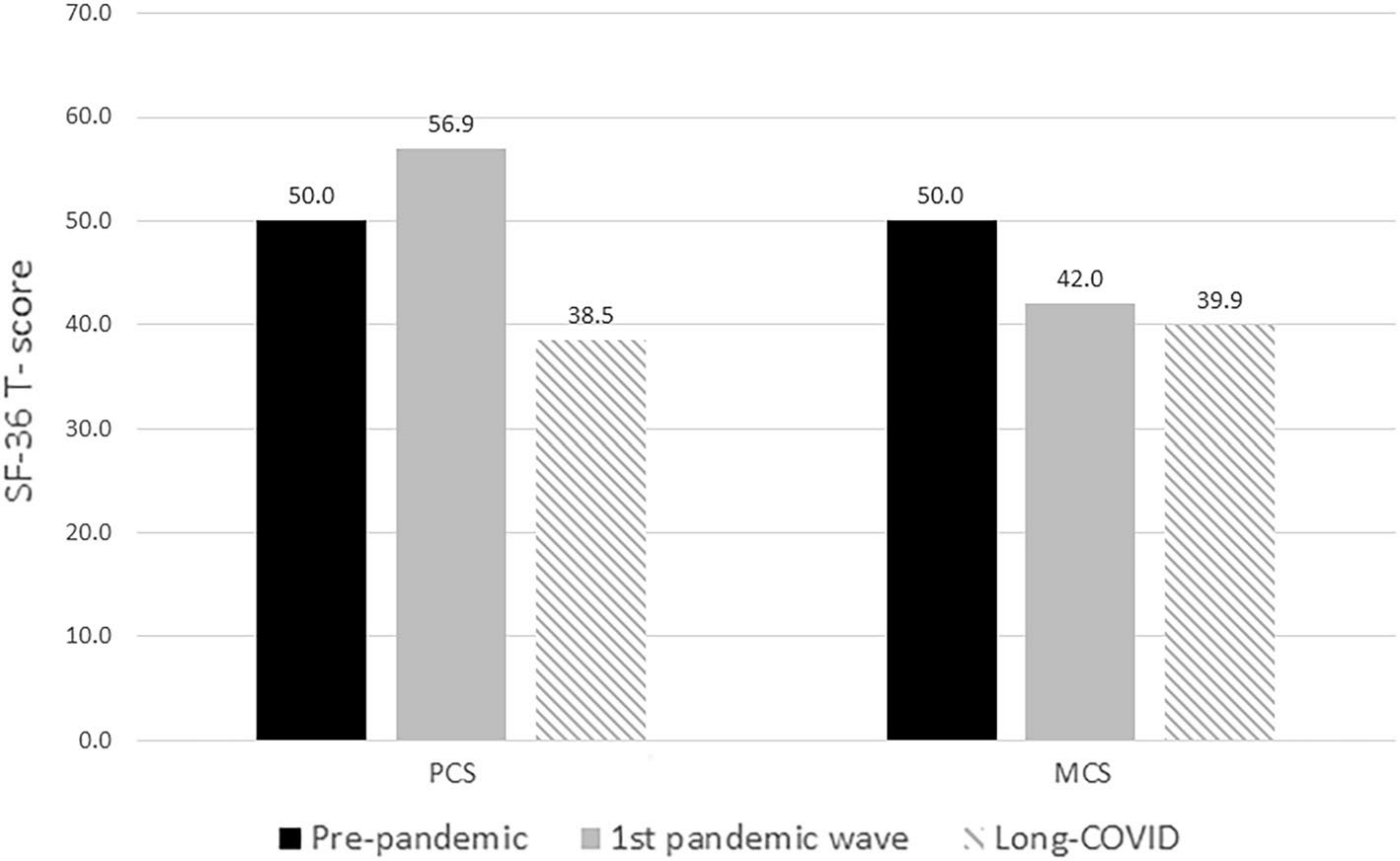
Mean PCS and MCS T-Scores of the SF-36. Figure shows mean physical component summary (PCS) and mental component summary (MCS) T-scores of the SF-36 in pre-pandemic Swiss general population (n = 1209), in the CoWell study population (n = 1581) and in Long-Covid patients (n = 107). Higher values indicate better health-related quality of life. PCS T-Scores are impaired in Long-Covid patients compared to participants in the CoWell study, whereas MCS T-Scores show a similar reduction compared to the pre-pandemic Swiss general population. Figure was created using Stata version 16.1 (StataCorp. 2019, Texas, TX, USA, https://www.stata.com/products).

Table 4 shows SF-36 mean (SD) scores of the eight health dimensions as well as PCS and MCS scores in our Long-Covid study and in the pre-pandemic Swiss general population. Additionally, PCS and MCS scores from the CoWell study are listed as well^31^. Physical and mental HRQoL was lower in patients suffering from Long-Covid syndrome than in the pre-pandemic Swiss general population and in the CoWell study. Mean (SD) values for PCS and MCS for our Long-Covid study cohort using the U.S. general population factor scoring coefficient were 38.79 (10.24) and 39.92 (11.52), respectively. A graphical comparison for physical and mental HRQoL of the three different populations is shown in Fig. 3. Subgroup analysis (Table S3, supplemental material) showed significantly lower scores for women in the physical functioning domain (*p* = 0.046), physical role limitation domain (*p* = 0.007), and bodily pain domain (*p* = 0.048). Participant characteristics of people during pandemic (CoWell study) are shown in Table S4 and T-Scores for PCS and MCS using U.S. general population and Swiss population factor scoring coefficient are shown in Table S6 of the supplemental material.

**Table 4:** SF-36 health domains, physical component summary (PCS) and mental component summary (MCS).

Health domain	Prepandemic swiss population (n = 1209), mean (SD)	CoWELL study (n = 1581), mean (SD)	Long-COVID cohort (n = 112), mean (SD)
Physical functioning	91.16 (17.01)	NA	63.72 (25.28)^a^
Role limitations (physical)	86.41 (20.60)	NA	28.15 (37.56)^b^
Bodily pain	74.58 (26.03)	NA	58.79 (28.47)
General health	75.64 (17.35)	NA	51.60 (19.12)^a^
Vitality/Energy	63.24 (17.22)	NA	29.22 (19.17)^a^
Social role functioning	85.84 (20.02)	NA	55.62 (30.02)^a^
Emotional role functioning	87.64 (19.22)	NA	52.08 (43.56)
Emotional well-being	75.02 (16.18)	NA	59.37 (19.63)^c^
PCS (T-Score)	50.0 (10.0)	56.9 (7.7)	38.5 (10.1)^d^
MCS (T-Score)	50.0 (10.0)	42.0 (10.7)	39.9 (11.5)^d^

*SD* standard deviation, *NA* not available.

^a^n = 109.

^b^n = 111.

^c^n = 108.

^d^n = 107.

## Discussion

To our knowledge, this is the first study that compares health-related quality of life in patients suffering from Long-Covid syndrome after initial mild to moderate COVID-19 infection with two reference groups (pre-pandemic and during the pandemic). The pandemic itself leads to impairments in mental aspects of quality of life, whereas people showed improvements in physical health during the first pandemic wave. On the contrary, Long-Covid syndrome has a considerable impact on physical health, while mental health seems not to be additionally impaired compared to individuals during the pandemic.

So far, studies investigating HRQoL in post-acute sequelae of SARS-CoV-2 infections mainly focused on patients who were hospitalized during the acute infection because data on developing Long-Covid syndrome in patients with initially mild or moderate disease severity gained attention much later. Pathophysiological mechanisms for developing long-lasting symptoms in patients with severe infections and mild cases might differ substantially and lead to different restrictions on health domains. Long-COVID syndrome is defined as ongoing symptoms after an acute infection disregarding the severity of the initial disease, which leads to a mixed patient population when assessing long-term outcomes. This study assessed HRQoL in patients suffering from Long-Covid symptoms after a mild to moderate infection in a Swiss cohort whose patient characteristics are comparable with previous studies in non-hospitalized Covid-19 patients^33,34^. The current study showed that patients with Long-Covid syndrome predominantly had problems performing their usual activities as well as issues with pain and anxiety, whereas mobility seemed not to be affected to the same extent. Patients showed markedly lower results in physical and mental HRQoL in the SF-36 questionnaire compared to HRQoL in the Swiss general population before the pandemic.

Since March 2020, Switzerland underwent several extraordinary measures to control the spread of SARS-CoV-2. Apart from entertainment and leisure establishments being closed, public and private events were prohibited. The effect of social distancing and self-isolation on quality of life is unclear, but several studies pointed out that social restrictions may lead to impairments of life quality, especially in regard to mental health^35,36^. To consider these effects, we compared our results with data on physical and mental health gathered in a Swiss cohort during the first lockdown in Switzerland (CoWell study). In this cohort, in which the proportion of female sex and mean age was similar to our cohort, individuals showed an increase in physical health (PCS), but there was a reduction in mental health (MCS) after the first infection wave. One might assume that during the first infection wave with its accompanying restrictions people were physically more active, especially as they had more time while being in home-office and home-workouts started to trend. However, a performed systematic review summarized that physical activity (PA) in healthy adults decreased during the pandemic^37^. One study analyzed data from activity trackers showed that people in Switzerland had a decrease in steps per day during lockdown^38^. Therefore, it could be that the appreciation of one’s own health during times of the pandemic might have contributed to increased self-perceived physical health. In comparison, Long-Covid patients showed a remarkable impairment in self-reported physical health while mental health was comparable. It should be noted that we started conducting this study during the second and third wave of the coronavirus, when restricting measures were reinstated. For that reason, it is difficult to assess the unique effect of Long-Covid symptoms on mental HRQoL. Although the restricting measures may not have been as affecting as in the first wave due to e.g. lesser fear of consequences of infection with the virus, a future comparison of the mental health recovery courses due to abandoned governmental restrictions would help to shed light on the unique effect of Long-Covid syndrome. Finally, compared to pre-pandemic results, PCS and MCS scores of our Long-Covid cohort considerably exceed the recommended 3-point minimal important difference and thus are of clinical relevance^39^. Consequently, the impairments in physical and mental functioning will lead to economic losses due to reduced working hours or sickness absence, but it remains to be determined to which extent.

In concordance with our results, Arnold et al.^40^ followed up patients for 8 to 12 weeks after a mild, moderate or severe acute SARS-CoV-2 infection and found a reduction across all health domains compared with age-matched population norms in the SF-36. This latter study showed similar results regarding PCS (mean (SD) of 36.0 (7.0)) and MCS (mean (SD) of 40.0 (7.0)) in patients with severe acute infection compared to our cohort including only patients who did not require hospitalization.

Beyond pandemic, several studies measured HRQoL in other chronic conditions using the SF-36 score. Patients suffering from fibromyalgia and ischemic heart disease showed similar scores for PCS compared to Long-Covid patients (mean (SD) of 38.6 (6.9) and 39.8 (9.9), respectively)^41,42^. Rheumatoid arthritis^41^ and chronic pain with major depressive disorder^43^ reached lower scores for PCS (Mean (SD) of 33.5 (6.4), 26.8 (8.4), and 25.5, respectively). Looking at MCS scores, patients with fibromyalgia and chronic pain with major depressive disorder reached lower scores, whereas patients with rheumatoid arthritis and ischemic heart disease scored higher values compared to our cohort. It seems that diseases with a relevant psychological aspect, as seen in depression and fibromyalgia, lead to greater impairments in mental health than Long-Covid. However, comparisons with other disease-specific populations must be treated with caution, as they might differ in sociodemographic characteristics, such as sex, age and educational background, which are known to be important factors associated with HRQoL.

EuroQol dimension responses showed results in line with the results from the SF-36 questionnaire with most impairments stated in the dimensions “usual activities”, “pain/discomfort” and “anxiety/depression” in our Long-Covid patients. Only a few previous studies reported similar findings in regards to the distribution of EuroQol dimension responses in patients with mild acute infections^44–46^. Similar to the results in our study, the dimension of “self-care” seems not to be affected in the study by Betschart et al.^44^ However, the heterogeneous distribution of EQ VAS values (visual score for total health) showed a broad variety not only in symptom burden but also in impact on quality of life with a slightly higher mean (SD) of 59.0 (20.3) in our study than the one found in a Belgian study performed 79 days after symptom onset (mean (SD) of 51 (19))^45^. The EQ-5D-5L index score from our study showed comparable results to patients 6 months after COVID-19 related acute respiratory distress syndrome (mean (SD) of 0.738 (0.21) and 0.705 (0.25), respectively)^47^.

To assess the impact of widely mentioned respiratory symptoms such as dyspnea (60% prevalence in our study cohort) on HRQoL we utilized the SGRQ. Respiratory symptoms had the greatest impact on patients PA. When comparing our results to those of a cross-sectional epidemiological study in COPD patients^48^, median scores for the SGRQ total and impact score component were similar to COPD patients with moderate disease severity (median (IQR) of 39.1 (26.5, 53.1) and 24.7 (13.5, 40.9), respectively). Moreover, patients with Long-Covid syndrome showed comparable impairments in PA with COPD patients with GOLD stages 2 to 3. Only the symptom score component was lower than in any GOLD stage group, indicating lower intensity and/or frequency of breathing symptoms in Long-Covid patients.

Interestingly, female sex was a predictor for low SGRQ activity scores, EQ-5D-5L index value as well as physical functioning, physical role limitations and bodily pain regarding the SF-36. It seems that especially in the field of PA females feel more limited. The reason for this has to be investigated, e.g. whether the difference between males and females regarding aspects of HRQoL is due to a higher symptom burden or subjective perceptions. In this study, a greater proportion of women (2/3) had equal or more than 6 symptoms compared to men (1/3), which might indicate that females are affected more intensely or broadly by Long-Covid symptoms. However, an alternative hypothesis could be the known gender difference in coping strategies during stressful events^49^.

This study has some limitations. First, there was no involvement of a control group investigating the same time point of the pandemic, thus results have to be interpreted with caution. However, the remarkable difference in physical health between the cohort of the CoWell study and the Long-Covid cohort is hard to explain only because of different time points of evaluation. Second, patients with a higher subjective symptom burden may fill out questionnaires more often, thus causing selection bias and thus not adequately reflecting the real symptom burden of a Long-Covid population.

## Conclusion

Long-lasting symptoms after a mild SARS-CoV-2 infection substantially affect health-related quality of life and therefore contribute considerably to the overall burden of the disease. Long-term surveillance of physical and mental health in affected patients is important to estimate the effect of Long-Covid syndrome on social and economic consequences.

## Supplementary Information

Supplementary Information.

## Data Availability

The dataset supporting the conclusion of this article is available from the corresponding author on reasonable request.

## Abbreviations

EQ-5D-5L: The EuroQol 5 Dimension 5 Level
HRQoL: Health-related quality of life
IQR: Interquartile range
MCS: Mental component summary
PA: Physical activity
PCS: Physical component summary
SD: Standard deviation
SF-36: The Short Form Health 36
SF-36v2: Short form health 36 questionnaire version 2
SGRQ: St. George’s Respiratory Questionnaire
VAS: Visual analog scale
WHO: The World Health Organization
