# Supplementary material for: Impaired health-related quality of life in long-COVID syndrome after mild to moderate COVID-19
Source: Sci Rep. 2023 May 12;13:7717. doi: 10.1038/s41598-023-34678-8 (PMC10175927; doi:10.1038/s41598-023-34678-8)
Supplement: Supplementary file 1 — Supplementary Information. [file 41598_2023_34678_MOESM1_ESM.docx]

**Impaired Health-related Quality of Life in Long-COVID syndrome after mild to moderate COVID-19**

*Supplemental material*

Stefan Malesevic^1,2^, Noriane A Sievi^2^, Patrick Baumgartner^1,2^, Katharina Roser^6^, Grit Sommer^7,8^, Dörthe Schmidt^3^, Florence Vallelian^4^, Ilijas Jelcic^5^, Christian F Clarenbach^1,2^, Malcolm Kohler^2^

1 University of Zurich, Faculty of Medicine, Zurich, Switzerland

2 University Hospital Zurich, Department of Pulmonology, Zurich, Switzerland

3 University Hospital Zurich, Department of Cardiology, Switzerland

4 University Hospital Zurich, Department of Internal Medicine, Switzerland

5 University Hospital Zurich, Department of Neurology, Switzerland

6 Department of Health Sciences and Medicine, University of Lucerne, Lucerne, Switzerland

7 Division of Pediatric Endocrinology, Diabetology and Metabolism, Department of Pediatrics, Inselspital, Bern University Hospital, University of Bern, Bern, Switzerland

8 Department of Biomedical Research, University of Bern, Bern, Switzerland

|  |  | Symptoms | | Activity | | | | Impacts | | | | Total | | |
| --- | --- | --- | --- | --- | --- | --- | --- | --- | --- | --- | --- | --- | --- | --- |
| *Sex* | *N* |  | | *N* |  | | *N* | |  | | *N* | |  |  |
| Female | 84 | 42.60 (29.8, 57.35) | p = 0.787 | 73 | 59.46 (41.40, 72.89) | **p = 0.005** | 76 | | 26.99 (15.58, 43.64) | p = 0.136 | 67 | | 41.07 (27.80, 58.18) | p = 0.091 |
| Male | 25 | 42.80 (26, 54.8) |  | 23 | 35.79 (17.12, 59.45) |  | 25 | | 22.33 (6.91, 35.36) |  | 22 | | 29.27 (25.19, 48.84) |  |
| *WHO classification* | *N* |  |  | *N* |  |  | *N* | |  |  | *N* | |  |  |
| Mild | 99 | 42.50 (27.00, 56.30) | p = 0.422 | 87 | 58.61 (29.31, 72.80) | p = 0.191 | 91 | | 25.65 (13.25, 40.89) | p = 0.416 | 81 | | 39.14 (26.17, 54.51) | p = 0.118 |
| Moderate | 10 | 50.95 (31.8, 67.30) |  | 9 | 66.19 (53.97, 79.67) |  | 10 | | 34.19 (15.81, 50.78) |  | 8 | | 58.07 (32.41, 65.39) |  |
| Values are presented as median (interquartile range). SGRQ: St. George’s Respiratory Questionnaire; WHO: world health organization. | | | | | | | | | | | | | | |

**Table S1. SGRQ component subgroup analysis.**

**Table S2. EQ index score subgroup analysis.**

| Subgroup | N | EQ index value, median (IQR) | p-value |
| --- | --- | --- | --- |
| *Sex* |  |  |  |
| Female | 84 | 0.788 (0.555, 0.887) | **p < 0.001** |
| Male | 26 | 0.909 (0.828, 0.909) |  |
| *WHO classification* |  |  |  |
| Mild | 99 | 0.806 (0.610, 0.909) | p = 0.764 |
| Moderate | 11 | 0.578 (0.427, 0.910) |  |
| EQ: Euroquol; WHO: world health organization. | | | |

**Table S3. SF-36 health domains subgroup analysis.**

| Subgroup | SF-36 Physical functioning | | Overall p-value |
| --- | --- | --- | --- |
| *Sex (female / male, %)* | **Female (N=84)** | **Male (N=25)** | **p = 0.046** |
| 0-49 points in physical functioning | 38.1 | 16.0 |  |
| 50 points in physical functioning | 2.4 | 0.0 |  |
| 51-100 points in physical functioning | 59.5 | 84.0 |  |
| *WHO classification (mild / moderate, %)* | **Mild (N=99)** | **Moderate (N=10)** | p = 0.213 |
| 0-49 points in physical functioning | 32.3 | 40.0 |  |
| 50 points in physical functioning | 2.0 | 0.0 |  |
| 51-100 points in physical functioning | 65.7 | 60.0 |  |
|  | **SF-36 Role limitations (physical)** | |  |
| *Sex (female / male, %)* | **Female (N=85)** | **Male (N=26)** | **p = 0.007** |
| 0-49 points in role limitations (physical) | 76.5 | 38.5 |  |
| 50 points in role limitations (physical) | 8.2 | 19.2 |  |
| 51-100 points in role limitations (physical) | 15.3 | 42.3 |  |
| *WHO classification (mild / moderate, %)* | **Mild (N=100)** | **Moderate (N=11)** | p = 0.433 |
| 0-49 points in role limitations (physical) | 67.0 | 72.7 |  |
| 50 points in role limitations (physical) | 12.0 | 0.0 |  |
| 51-100 points in role limitations (physical) | 21.0 | 27.3 |  |
|  | **SF-36 Bodily pain** | |  |
| *Sex (female / male, %)* | **Female (N=86)** | **Male (N=26)** | **p = 0.048** |
| 0-49 points in bodily pain | 41.9 | 11.5 |  |
| 50 points in bodily pain | 0.0 | 0.0 |  |
| 51-100 points in bodily pain | 58.1 | 88.5 |  |
| *WHO classification (mild / moderate, %)* | **Mild (N=101)** | **Moderate (N=11)** | p = 0.555 |
| 0-49 points in bodily pain | 34.7 | 36.4 |  |
| 50 points in bodily pain | 0.0 | 0.0 |  |
| 51-100 points in bodily pain | 65.3 | 63.6 |  |
|  | **SF-36 General Health** | |  |
| *Sex (female / male, %)* | **Female (N=83)** | **Male (N=26)** | p = 0.721 |
| 0-49 points in general health | 46.9 | 38.5 |  |
| 50 points in general health | 4.9 | 3.9 |  |
| 51-100 points in general health | 48.2 | 57.8 |  |
| *WHO classification (mild / moderate, %)* | **Mild (N=99)** | **Moderate (N=10)** | p = 0.213 |
| 0-49 points in general health | 46.5 | 30.0 |  |
| 50 points in general health | 5.0 | 0.0 |  |
| 51-100 points in general health | 48.5 | 70.0 |  |
|  | **SF-36 Vitality/Energy** | |  |
| *Sex (female / male, %)* | **Female (N=84)** | **Male (N=25)** | p = 0.579 |
| 0-49 points in vitality/energy | 83.3 | 68.0 |  |
| 50 points in vitality/energy | 3.6 | 16.0 |  |
| 51-100 points in vitality/energy | 13.1 | 16.0 |  |
| *WHO classification (mild / moderate, %)* | **Mild (N=98)** | **Moderate (N=11)** | p = 0.098 |
| 0-49 points in vitality/energy | 79.6 | 81.8 |  |
| 50 points in vitality/energy | 6.1 | 9.1 |  |
| 51-100 points in vitality/energy | 14.3 | 9.1 |  |
|  | **SF-36 Social role functioning** | |  |
| *Sex (female / male, %)* | **Female (N=84)** | **Male (N=25)** | p = 0.494 |
| 0-49 points in social role functioning | 40.5 | 20.0 |  |
| 50 points in social role functioning | 14.3 | 24.0 |  |
| 51-100 points in social role functioning | 45.2 | 56.0 |  |
| *WHO classification (mild / moderate, %)* | **Mild (N=98)** | **Moderate (N=11)** | p = 0.556 |
| 0-49 points in social role functioning | 34.7 | 45.5 |  |
| 50 points in social role functioning | 18.4 | 0.0 |  |
| 51-100 points in social role functioning | 46.9 | 54.5 |  |
|  | **SF-36 Emotional role functioning** | |  |
| *Sex (female / male, %)* | **Female (N=86)** | **Male (N=26)** | p = 0.931 |
| 0-49 points in emotional role functioning | 50.0 | 53.9 |  |
| 50 points in emotional role functioning | 0.0 | 0.0 |  |
| 51-100 points in emotional role functioning | 50.0 | 46.1 |  |
| *WHO classification (mild / moderate, %)* | **Mild (N=101)** | **Moderate (N=11)** | p = 0.262 |
| 0-49 points in emotional role functioning | 52.5 | 27.3 |  |
| 50 points in emotional role functioning | 0.0 | 0.0 |  |
| 51-100 points in emotional role functioning | 47.5 | 72.7 |  |
|  | **SF-36 Emotional well-being** | |  |
| *Sex (female / male, %)* | **Female (N=83)** | **Male (N=25)** | p = 0.096 |
| 0-49 points in SF-36 emotional well-being | 36.2 | 28.0 |  |
| 50 points in SF-36 emotional well-being | 0.0 | 0.0 |  |
| 51-100 points in SF-36 emotional well-being | 63.8 | 72.0 |  |
| *WHO classification (mild / moderate, %)* | **Mild (N=98)** | **Moderate (N=10)** | p = 0.485 |
| 0-49 points in SF-36 emotional well-being | 34.7 | 30.0 |  |
| 50 points in SF-36 emotional well-being | 0.0 | 0.0 |  |
| 51-100 points in SF-36 emotional well-being | 65.3 | 70.0 |  |
| SF36: short form health 36; WHO: world health organization. | | | |

| Table S4. CoWell-study patient characteristics. | |
| --- | --- |
| **Sex** | |
| Men | 386 (24) |
| Women | 1195 (76) |
| **Age, years (mean (SD))** | 45.4 (14.7) |
| **Age at survey, years (mean (SD))** | |
| 18-25 | 124 (8) |
| 26-35 | 449 (28) |
| 36-45 | 375 (24) |
| 46-55 | 318 (20) |
| 56-65 | 202 (13) |
| >=66 | 113 (7) |
| **Highest educational level** | |
| Compulsory or vocational schooling | 305 (19) |
| Upper secondary education | 252 (16) |
| University education | 972 (61) |
| Unknown | 52 (3) |
| **Language of questionnaire** | |
| German | 1431 (91) |
| French or Italian | 150 (9) |
| **Employment status** | |
| Employed or in education | 1393 (88) |
| Other | 154 (10) |
| Unknown | 34 (2) |
| **Number of children <14 years in household** | |
| None | 1164 (74) |
| One or more | 417 (26) |
| Values are presented as N (%) unless otherwise stated. | |

**Table S5. EQ-5D-5L distribution of dimension responses.**

| Dimension | N (%) |
| --- | --- |
| Mobility |  |
| *No problems* | 51 (46.4) |
| *Slight problems* | 26 (23.6) |
| *Moderate problems* | 22 (20.0) |
| *Severe problems* | 11 (10.0) |
| *Unable to walk about* | 0 (0) |
| Self-care |  |
| *No problems* | 96 (87.3) |
| *Slight problems* | 10 (9.1) |
| *Moderate problems* | 4 (3.6) |
| *Severe problems* | 0 (0) |
| *Unable to wash or dress* | 0 (0) |
| Usual activities |  |
| *No problems* | 15 (13.6) |
| *Slight problems* | 35 (31.8) |
| *Moderate problems* | 31 (28.2) |
| *Severe problems* | 18 (16.4) |
| *Unable to do usual activities* | 11 (10.0) |
| Pain/discomfort |  |
| *No pain/discomfort* | 14 (12.7) |
| *Slight pain/discomfort* | 30 (27.3) |
| *Moderate pain/discomfort* | 42 (38.2) |
| *Severe pain/discomfort* | 23 (20.9) |
| *Extreme pain/discomfort* | 1 (0.9) |
| Anxiety/depression |  |
| *Not anxious/depressed* | 24 (21.8) |
| *Slightly anxious/depressed* | 39 (35.5) |
| *Moderately anxious/depressed* | 30 (27.3) |
| *Severely anxious/depressed* | 15 (13.6) |
| *Extremely anxious/depressed* | 2 (1.8) |

**Table S6. SF-36 mean (SD) T-Scores for PCS and MCS.**

|  | PCS | MCS |
| --- | --- | --- |
| Switzerland prepandemic (using Swiss factor score coefficient) | 50.0 (10.0) | 50.0 (10.0) |
| Switzerland during 1^st^ pandemic wave (using Swiss factor score coefficient) | 56.9 (7.7) | 42.0 (10.7) |
| Switzerland during 1^st^ pandemic wave (using USA factor score coefficient) | 57.5 (6.0) | 42.8 (9.0) |
| Long-Covid patients (using Swiss factor score coefficient) | 38.5 (10.1) | 39.9 (11.5) |
| Long-Covid patients (using USA factor score coefficient) | 38.8 (10.2) | 39.9 (11.5) |
| Values are presented as mean (SD). PCS: physical component summary; MCS: mental component summary. | | |
